# SAFoldNet: A Novel Tool for Discovering and Aligning Three-Dimensional Protein Structures Based on a Neural Network

**DOI:** 10.3390/ijms241914439

**Published:** 2023-09-22

**Authors:** Denis V. Petrovskiy, Kirill S. Nikolsky, Vladimir R. Rudnev, Liudmila I. Kulikova, Tatiana V. Butkova, Kristina A. Malsagova, Arthur T. Kopylov, Anna L. Kaysheva

**Affiliations:** Institute of Biomedical Chemistry, 119121 Moscow, Russia; petro2017@mail.ru (D.V.P.); glucksistemi@gmail.com (K.S.N.); v.r.rudnev@gmail.com (V.R.R.); likulikova@mail.ru (L.I.K.); t.butkova@gmail.com (T.V.B.); a.t.kopylov@gmail.com (A.T.K.); kaysheva1@gmail.com (A.L.K.)

**Keywords:** protein conformation, protein structure, protein motif, protein domain, neural network, structural alphabet

## Abstract

The development and improvement of methods for comparing and searching for three-dimensional protein structures remain urgent tasks in modern structural biology. To solve this problem, we developed a new tool, SAFoldNet, which allows for searching, aligning, superimposing, and determining the exact coordinates of fragments of protein structures. The proposed search and alignment tool was built using neural networking. Specifically, we implemented the integrative synergy of neural network predictions and the well-known BLAST algorithm for searching and aligning sequences. The proposed method involves multistage processing, comprising a stage for converting the geometry of protein structures into sequences of a structural alphabet using a neural network, a search stage for forming a set of candidate structures, and a refinement stage for calculating the structural alignment and overlap and evaluating the similarity with the starting structure of the search. The effectiveness and practical applicability of the proposed tool were compared with those of several widely used services for searching and aligning protein structures. The results of the comparisons confirmed that the proposed method is effective and competitive relative to the available modern services. Furthermore, using the proposed approach, a service with a user-friendly web interface was developed, which allows for searching, aligning, and superimposing protein structures; determining the location of protein fragments; mapping onto a protein molecule chain; and providing structural similarity metrices (expected value and root mean square deviation).

## 1. Introduction

At present, many programs and libraries are available for structural searches and alignments in the databases of genomic or amino acid sequences. A number of tools, such as BLAST (https://blast.ncbi.nlm.nih.gov/, accessed on 3 June 2023) [1], which is the most popular tool among molecular biologists and bioinformaticians, search for protein and nucleic acid homologs based on primary protein sequences or fragments. These tools typically use graph matching and dynamic programming techniques to identify and align Cα atoms in the primary chain or secondary structures [2].

However, in nature, the three-dimensional structure of a protein and its spatial topology are more conserved than its amino acid sequence; therefore, the search for and comparison of structures based on their three-dimensional topologies are important elements for understanding the functions of proteins and classifying protein families. This task is particularly urgent given the recent significant increases in the number of three-dimensional protein structures available in various databases (Protein Data Bank (PDB), AlphaFold, and CATH, among others).

To solve the problem of searching for and aligning three-dimensional structures, predictors of the various properties of protein structure are employed, including position invariant descriptors obtained from spherical harmonics and Zernike polynomials [3,4] based on spherical polar Fourier representations [5,6], among others. In addition, an approach to describing the three-dimensional structure of a protein is to determine the sequences of repetitive classic secondary structures, such as α-helices, β-sheets, and coils [7]; however, this approach leaves approximately 50% of the protein structures undefined. A more accurate description of three-dimensional structures can be provided using prototypes, which approximate the local conformation of the three-dimensional protein structure. Prototypes are defined on the basis of fragments of consecutive amino acids in polypeptide chains, and a set of defined prototypes constitutes a library of local conformations of protein structures, the so-called structural alphabet (SA). The first major structural alphabet was published in 1989 by Unger et al. [8]; in that study, the authors demonstrated that combining local structure prototypes can correctly approximate a protein structure. Subsequently, many structural alphabets were developed to approximate local protein structures, which differ in terms of the properties used to describe the protein backbone (atomic coordinates, distances, and torsion angles); methods used to determine these properties (clustering algorithms, Kohonen maps, and artificial neural networks); and number of prototypes or letters. Each SA is defined by a set of prototypes and the length of the amino acid sequence for each prototype. One of the most popular and commonly used SAs is Protein Blocks, which comprises 16 prototypes [9,10].

The present article introduces a novel tool, SAFoldNet, which employs an amino acid sequence-independent algorithm for searching and aligning protein structures using a neural network and a SA comprising 23 prototypes described earlier [11]. A neural network is implemented to encode the three-dimensional protein structures in SA sequences, and the well-known BLAST+ is used to search, align, and evaluate the statistical significance of matches.

In addition to the high speed of searching for structures of arbitrary lengths, the tool accurately determines the coordinates of a given structure in the detected protein, performs pairwise alignment and superposition of the detected structures, and provides the expected value (E-value) metrices of the statistical significance of a structural similarity and root mean square deviation (RMSD). In addition, the service provides key characteristics useful to the user for each identified protein structure, including taxonomic affiliation, name, and biological function of the protein; PDB ID; and experimental parameters (resolution, method, and link to the experiment in PDB). The SAFoldNet service is available to users at https://psskb.org/similar-search (accessed on 3 June 2023).

## 2. Results

### 2.1. SAFoldNet

We developed SAFoldNet to swiftly search for protein structures and their fragments in PDB using neural networking and BLAST. The neural network encodes three-dimensional protein structures in a SA sequence. The developed tool uses a multistage process involving a preprocessing stage using a neural network to represent the geometry of the protein structures in the SA sequence, a search stage to generate a set of candidates, and a refinement stage to calculate the structural alignment and overlay and determine the location of the structure or fragment in a protein.

The present tool has a convenient web interface that allows for the visualization and evaluation of search results in real time.

### 2.2. Evaluation

To assess the accuracy and sensitivity of the algorithm, we used reference datasets SCOP and CATH [12] to generate two sets of test data. From SCOP version 1.75, we obtained a nonredundant set of 3537 proteins with no more than 20% identity. Another dataset was CATH-SCOP, which included protein domain sequences (n  =  1754) obtained from CATH v.4.3. apart from those of the SCOP test dataset. Data were percolated using a maximum sequence identity of 20%, and the final testing datasets are available here [13].

The SCOP superfamily is treated like true positives (TP) when used as a hit model for proteins in a query. Domains from another SCOP superfamily are considered false positives (FP). Although it is possible that homology relationships exist between some SCOP superfamilies (one rule for identifying such homologies was established by Julian Gough [14]), we ignored such potential matches in our assessment.

Matches between the query superfamily and the search results for the CATH dataset were considered in the same way. For assessment, the relationship between the E-value of the first hit and the number of correct and false superfamily assignments was calculated for the test datasets. The assessment also included a coverage indicator, which is defined as TP/Total, where “TP” is the number of correct matches, and “Total” is the total number of structures in the superfamily.

**Figure 1:**
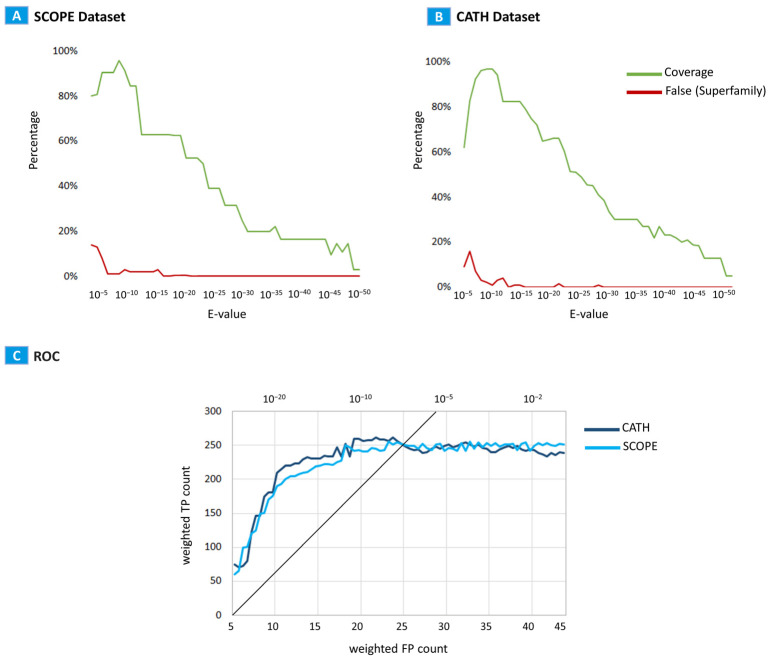
Superfamily level homology detection benchmark across database searches of the SCOP and CATH validation datasets. (**A**) The relationship between the E-values and the percentages of coverage values of the function assignments True (green) and False (red) for the SCOPE dataset. Coverage = (True positive)/Total in the superfamily. The coverage values of the function assignments are 81.16% (<10^−5^), 91.71% (<10^−10^), 92.31% (10^−15^), 75.22% (<10^−20^), and 58.97 (<10^−25^). (**B**) The relationship between the E-values and the percentages of coverage values of the function assignments True (green) and False (red) for the CATH dataset. The coverage values of the function assignments are 71.16%(<10^−5^), 93.54% (<10^−10^), 93.07% (10^−15^), 78.17% (<10^−20^), and 61.01% (<10^−25^). (**C**) ROC plot for weighted FP versus weighted TP counts up to the E-values. Each FP or TP is weighted by 1/(number of the other domains in the query superfamily).

The ROC curve plots the TP and FP values as certain threshold changes, where larger areas under the ROC curve indicate better performances. We plotted the ROC curve using the expected value (E-value) as the threshold. TP and FP estimates were weighted by 1/(number of other homologs that belong to the query superfamily) to prevent bias in the estimates by large protein superfamilies (Figure 1).

The optimal performance for these datasets is achieved in the E-value range from 10^−5^ to 10^−15^ (Figure 1). As the E-value increases, plenty of structures with fragmentary structural similarities appear in the search results. However, these structures do not belong to the superfamily, because the maximum number of structures in the search results was limited to 2048; thereafter, the coverage indicator was noticeably reduced. As the E-value decreases, the accuracy of structural matches increases, and the number of detected structures decreases. For the very small E-values, only the requested structure and their full homologues are present in the search results.

**Figure 2:**
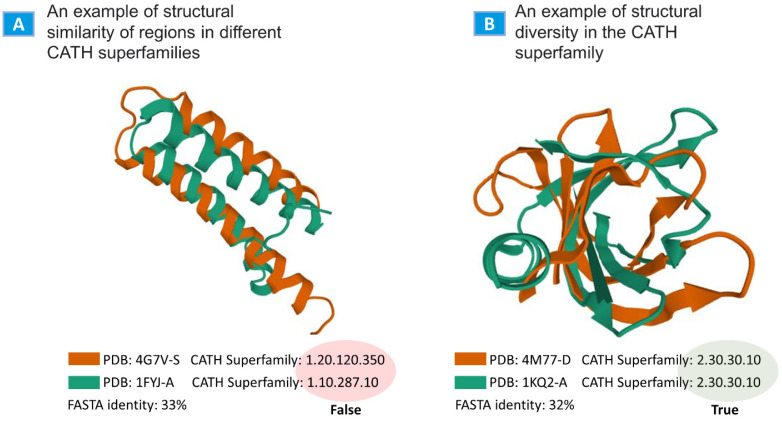
Examples from the validation results. (**A**) An example showing the structural similarity of protein chains belonging to different CATH superfamilies. Query: PDB: 1FYJ chain A (1.10.287.10). The search results contain PDB 4G7V S. The structure is similar to the query, but it belongs to the 1.20.120.350 family. E-value = 2 × 10^−4^. (**B**) Protein regions with a weak structural similarity but belong to the same CATH superfamily. Query: PDB: 2KQ2 chain A (2.30.30.10). The search results include PDB 4M77-D, the superfamily of which matches the query.

Two intriguing examples from the testing results were obtained. One case displayed two protein regions with good structural similarity but that belonged to different CATH superfamilies (Figure 2A), and such cases were marked as FP (false positive) in the validation results. Another case (Figure 2B) included results where, visually, there were no structural similarities, but nevertheless, the structures belonged to the same CATH superfamily.

Additionally, to evaluate the quality of the developed tool, we conducted a comparative analysis and evaluation of the search accuracy with other popular services: 3D-BLAST (http://3d-blast.life.nctu.edu.tw/, accessed on 3 June 2023), a search tool with the same SA implemented on the PDB website (3D-structure search) (https://www.rcsb.org/, accessed on 3 June 2023), and the well-known search service PDBeFold (https://www.ebi.ac.uk/msd-srv/ssm/, accessed on 3 June 2023).

A comparison was performed for three structures representing a family of small proteins from different domains: ubiquitin, immunoglobulin, and PDZ. The total number of structures detected was analyzed, and the correspondence of the detected structures to the original domain was assessed according to CATH classification. The CATH superfamilies for search results were assigned using the FunFHMMer web server (https://www.cathdb.info/search/by_sequence, accessed on 3 June 2023). The results of the comparison are presented in Table 1.

**Figure 3:**
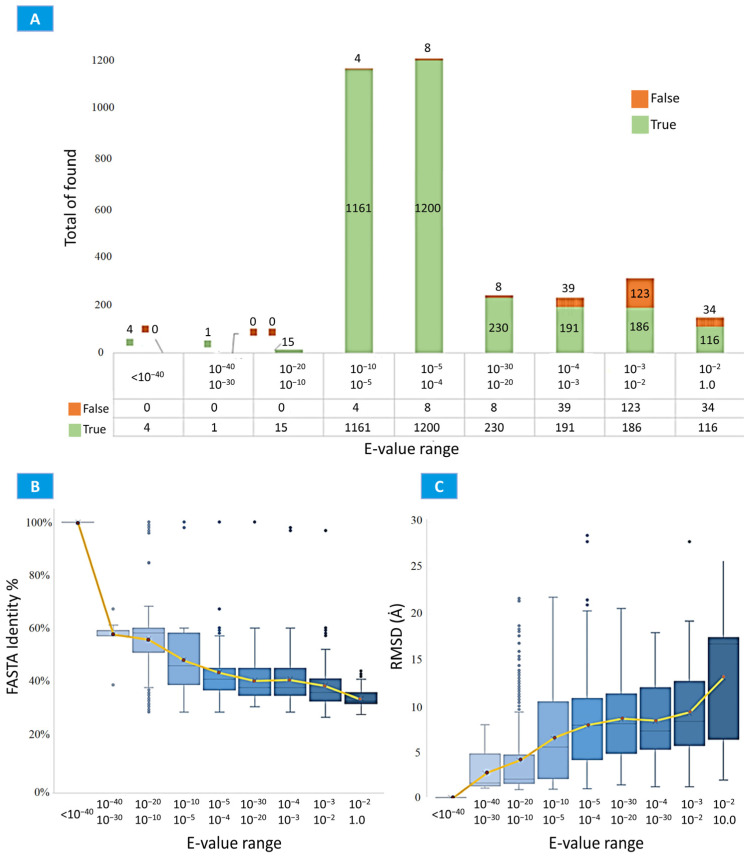
The relationship between the E-values and the correctly assigned CATH superfamily for ubiquitin, Pdz3 domain, and immunoglobulin. In general, the results correlate with the conclusions obtained from testing SAFoldNet on the validation datasets. The optimal results are achieved with E-values in the range 10^−5^–10^−15^ (**A**). Identity of FASTA in the search results with respect to the requested structure based on the E-values (**B**). RMSD between the requested and search structures as a function of the E-value shows that, as the E-value increases, the structural similarity between the query and the result decreases (**C**).

As shown in Table 1, the service indicators of SAFoldNet surpassed those of 3D-BLAST and RCSB PDB Search in terms of the number of correctly identified structures and were comparable to those of PDBeFold in terms of the search volume and quality. Simultaneously, in the SAFoldNet service, a rather large cutoff threshold of statistical significance in the search results was set at E-value = 1.0. In the graph of the association between the E-value and percentage of correctly defined structures in Figure 3, the majority of the errors fall in the rightmost range, corresponding to high E-values.

For the considered set of structures, if the E-value cutoff was set to 10^−3^, then the accuracy of the CATH family assignment was 97.9% and the number of correctly identified structures was 2802, which exceeded the results of the other services selected for comparison. Unfortunately, we cannot provide a universal recipe for selecting an optimal E-value cutoff, which would provide high, close to 100%, search accuracy while providing the maximum number of precisely defined structures in search results, which primarily depends on the size of the structure and purpose of the search. As such, for short structures with dimensions of 30 AA and fewer, an E-value cutoff of 0.7–1.0 is expected to be the optimal value; meanwhile, the cutoff range of 10^−3^–10^−4^ may be optimal for structures with an average size of 30–100 AA and 10^−5^ for even larger structure of >100 AA.

In addition, we examined the association between the statistical significance and amino acid sequence identity between the queried and detected structures in the search results: plots of FASTA distributions versus E-values and of RMSD distributions versus E-values (Figure 3B,C). 

**Figure 4:**
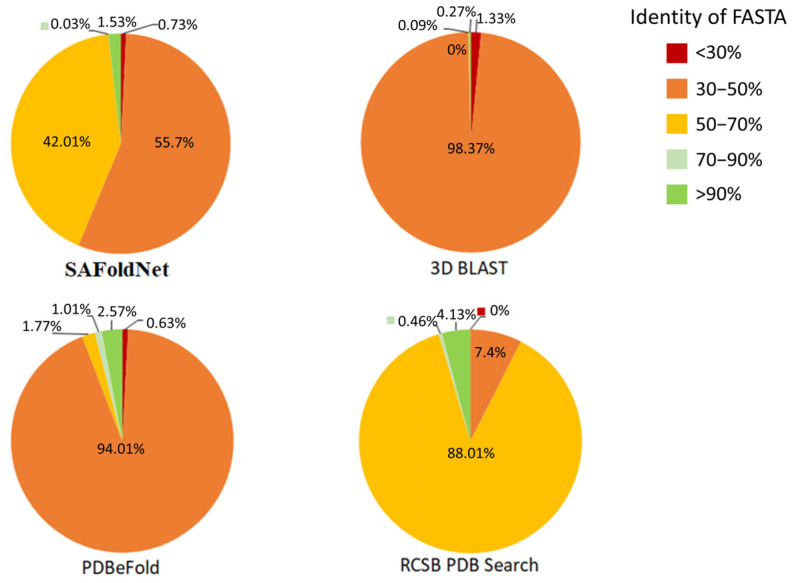
Distribution of amino acid sequence identity (identity of FASTA%) in the search results relative to the original structure.

As expected, with an increase in the value of the statistical significance, the variability of the set of detected structures relative to the initial one increased, both in terms of the amino acid sequence and three-dimensional conformations (Figure 4).

From these diagrams, each service presents characteristic features in terms of the proportions of this distribution; apparently, such a distribution can be attributed to the features of the search algorithms.

Furthermore, we considered the 3D BLAST service, which uses the same SA of 23 prototypes as the developed service, although its search accuracy indicators are significantly lower. We analyzed the base of the structural sequences of the 3D BLAST service and determined that, although all protein structures from the PDB were encoded in the same alphabet, the differences between the structural sequences in 3B BLAST and the base of the developed service exceeded 40%. In other words, the same regions of the protein were encoded by different elements of the SA. Therefore, our neural network could approximate protein structures much more accurately and display them as a SA sequence as opposed to the method used in the 3D BLAST service.

**Figure 5:**
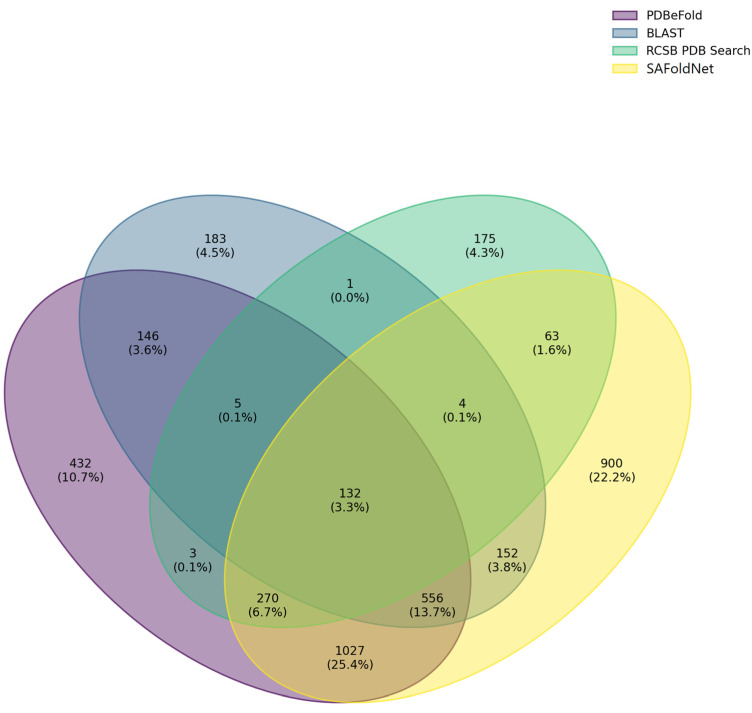
Venn diagram of the similarity of the search results for the protein structures among the four tools (PDBeFold, Blast, RCSB PDB Search, and SAFoldNet).

In addition, a comparative analysis was performed to determine the uniqueness of the correctly defined protein structures for each service, and the intersection of their sets was determined (Figure 5).

From this comparison, all sets showed rather large areas of intersection; nevertheless, each search service had its own unique subset of structures; relative to our service, the size of the unique subset of structures detected was 22%. Therefore, our service can be successfully used as an independent search tool or as an excellent addition to the repertoire of existing services for searching, classifying, and analyzing the evolution of protein structures.

## 3. Discussion

**Figure 6:**
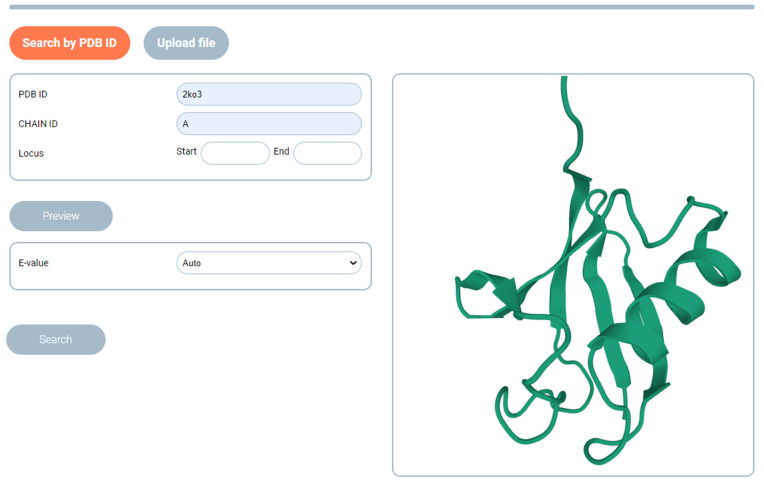
Search page for a service based on the PDB ID (with indication of the locus of interest, if necessary) or a file in the “.pdb” format.

SAFoldNet implements an understandable web interface for a wide range of users, including biochemists, molecular biologists, and structural biologists. The starting page of the tool prompts the user to search for a set of structures that are similar in conformation or their individual motifs. Both a protein of arbitrary length (PDB ID, indicating the locus of interest and protein chain) and its fragment in the form of a PDB file (Figure 6) can be submitted to the service input. 

The user can visualize the starting structure in various views (ribbon, backbone, and surface views). When starting a search, the user can limit the search results using statistical significance (E-value).

**Figure 7:**
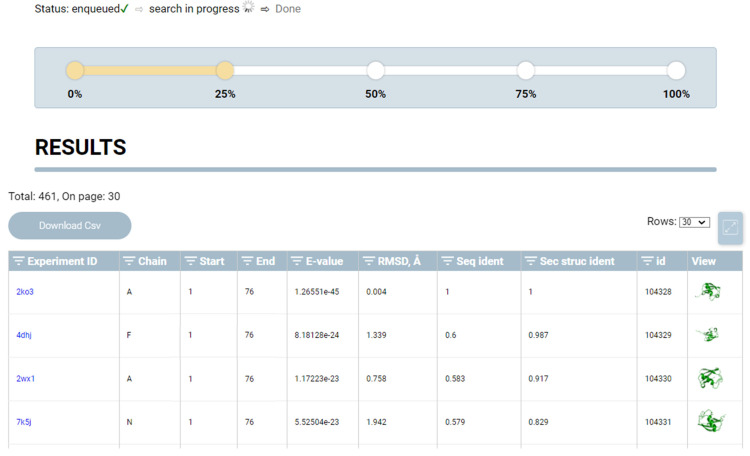
Search results page. It displays information on the starting structure of the search, status of the search, and search results.

The search results page presents basic information regarding the starting structure in a tabular form: protein data (UniProt ID, protein name, taxonomic affiliation, and biological process); experimental structure data (PDB reference, PDB ID, resolution, and experiment type); and protein structural information (locus, number of amino acid residues, and amino acid sequence of the motif in FASTA format) (Figure 7).

The search is performed in real time, and the search progress indicator bar displays the current search status and progress. The search result block is organized in a tabular form and presents data on the number of similar protein structures identified, E-value, RMSD relative to the start structure, amino acid sequence (Seq ident), and secondary structure (Sec struc ident) alignment results. The user can upload the search results in “. csv” format. In addition to the presented CSV data, the table includes information on the secondary sequence of the protein.

**Figure 8:**
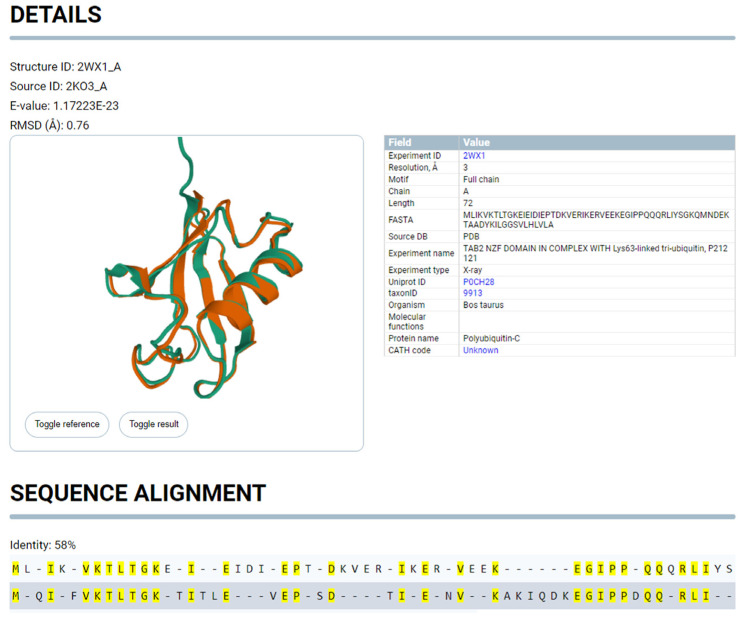
Individual card page of the detected protein structure.

Each structure from the search results can be additionally analyzed by the user when going through the “View” link to the graphical representation of the protein structure (Figure 8), which allows for a visual assessment of the similarity of the detected and starting structures.

The sequence and secondary structure identity blocks on the graphics card display the alignment results of the identified and start structures in terms of their amino acid sequence and secondary structure, respectively.

Unlike analogs, our service provides clear information regarding the localization of the detected structure in the protein chain and defines clear boundaries of the locus. The graphical representation helps the user evaluate the quality of the identification.

## 4. Materials and Methods

### 4.1. SA

The SA described in [11] was used as the basis. The alphabet is a profile of protein fragments with five amino acid residues; accordingly, the structure of a protein of L-amino acid residues is described by a sequence of a SA of length L-4 prototypes. These 23 structural prototypes represent the profiles of most three-dimensional protein fragments and can be roughly divided into five categories: helix–helix alphabets (A, Y, B, C, and D); helix-like–helix alphabets (G, I, and L); the strand–thread alphabet (E, F, and H); the strand-like thread alphabet (K and N); and others (S, T, V, W, X, M, P, Q, R, and Z). View segments in the same category are similar [11].

### 4.2. Neural Network Architecture

The architecture of the encoder for the vector representation of structures was based on a modified version of PSSNet developed by the authors earlier [15]. This network has been proven to solve the problem of the classification and segmentation of super-secondary structures (SSS). The network variant was modified by adding output Geometric Vector Perceptron layers to the original model to estimate the class of elements of SA and secondary structure assignments from STRIDE. In addition to the structural alphabet (SA) sequence, the network also predicts the 8-element STRIDE (secondary structure assignment) alphabet, which also appears in search results [16].

### 4.3. Neural Network Flow

The data transformation algorithm and network architecture are described in detail elsewhere [15]. The essence of the algorithm is described below:

1. From the array of coordinates, a graph is formed, each vertex of which corresponds to the Cα-atom of the primary protein chain, connected by edges with Cα-atoms (KNN graph, k = 32). Each edge and vertex of the graph contain scalar and vector features that describe the orientation of the amino acid residue in three-dimensional Euclidean space.
2. Signs of the graph vertex comprise torsion angles, single vectors that determine the direction of the previous and subsequent C and Cα atoms of the primary chain, single vectors that determine the direction of the side chain (direction to the Cβ-atom), and codes of the amino acid sequence of the protein.
3. The graph edge features comprise a single vector that defines the direction of the edge, distance between graph vertices encoded using Gaussian radial basis functions, and edge position code encoded using a positional sinusoidal encoder.
4. All graph features are input into the network, which comprises two interconnected blocks: an encoder, which generates a hidden feature representation, and a decoder, which converts the hidden feature representation into SA elements. A negative log likelihood *loss* function is used to calculate the network loss function.

**Figure 9:**
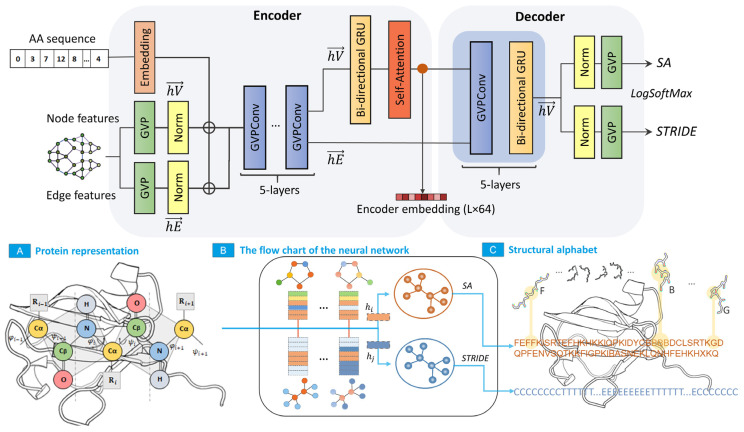
Network architecture and diagram of the data conversion process. (**A**) Representation of the three-dimensional structure of a protein as a feature graph. (**B**) Encoding three-dimensional representation using a neural network and matching graph nodes to structural alphabet elements. (**C**) Structural alphabet sequence for a three-dimensional structure.

A schematic representation of the network structure and data processing steps are shown in Figure 9.

### 4.4. Datasets

The training, test, and validation sets were retrieved from the PDB, encoded using the selected SA. Before training the network, the original set was cleaned, and structures with a short length (<16 AA) and low resolution (>3.5Å) were excluded. The resulting dataset, with a volume of ~90k copies (PDB chains), was divided into the training, test, and validation sets. The dimensions of these sets are listed in Table 2.

### 4.5. Network Training

The network training lasted 46 epochs. During training, the mechanism of automatic stopping of the learning process (early stopping) was applied when the accuracy indicator reached a plateau. The hyperparameters used in model training are presented in Table S1 (Supplementary Materials). Because the set presented an acceptable size for machine learning tasks, additional data augmentation was not performed.

Graphs of changes in the indicators depending on the learning epoch are shown in Figure S1 (Supplementary Materials).

### 4.6. Algorithm for Searching for Structures with Similar Conformations

To search for a set of structures similar in conformation or their individual motifs, both a protein of arbitrary length and its fragment in the form of a PDB file can be input into the service.

1. From the requested structure submitted to the input of the network, the features are extracted, and a structure graph (featurization) is created.
2. The network encoder block generates a vector of hidden feature representation (embedding) for the requested structure. The input for the search can be either a full chain (as deposited in the PDB) or a fragment.
3. The block decoder network (decoder) generates a sequence of SA for the requested structure.
4. Using the programing interface provided by the BLAST package, areas of similarity between sequences of the SA of the requested structure in the SA database are determined, and the statistical significance of similarity (E-value) is calculated. At the output, an array is formed comprising the indices of PDB files, elements of the identified areas, and value of the statistical significance index. E-value is a parameter describing the number of matches that can be “expected” or detected by chance when searching or browsing the sequence database. This value decreases exponentially as the number of matches increases. Essentially, the E-value describes random background noise. For instance, an E-value of 1 may imply that, in a SA database, one would detect a match simply by chance by looking at the content of the database. The lower the E-value or the closer it is to zero, the more “significant” the match. The E-value can also be used as a convenient metric to create a threshold of significance in structural similarity searches and in search result reports. The methodology for calculating this indicator is presented in detail elsewhere [17].
5. Next, the BLAST search results are sequentially processed, and each protein from the search results containing elements of the requested sequence is passed through the network encoder block (encoder) to obtain a latent representation vector that aggregates the geometric characteristics of the protein. This vector, together with the vector (embeddings) of the desired structure, is used to determine the exact location of the structure in the protein from the search results (this is relevant if a protein fragment is input and it is necessary to determine its exact location in the search results). The location is determined using a matrix of pairwise distances between embeddings, and the region with the smallest distance determines the location. This approach allows for accurately determining the position of a structure in the composition of large proteins.
6. After the location is determined, the desired and detected structures are superimposed using the Kabsh algorithm [18], and the RMSD between the Cα atoms of the structures is calculated.
7. At the output, a report is generated containing the detected structures, dropped and superimposed on the original structure, and indicators:
  - Statistical significance (E-value);
  - RMSD of atomic positions;
  - Structural coordinates within the protein;
  - FASTA matches (Sequence identity);
  - Secondary structure identity.

**Figure 10:**
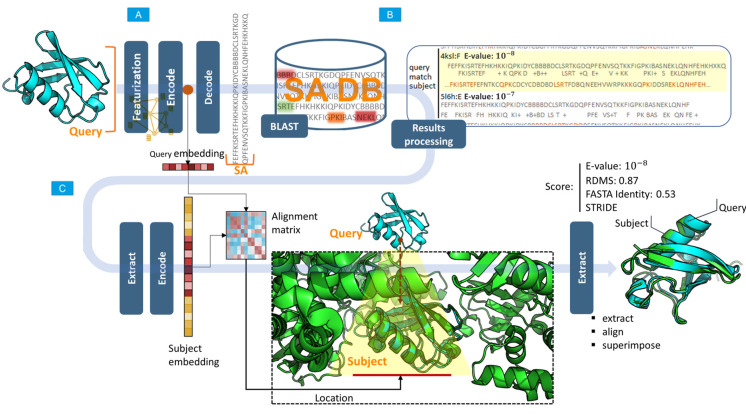
Scheme of the search process. (**A**) Representing a query as a structural alphabet sequence using a neural network. (**B**) Database search for structural sequences. (**C**) Results processing: location determination, overlay, and report generation.

An explanatory diagram of the search process is shown in Figure 10.

## 5. Conclusions

We introduce the novel tool SAFoldNet for the searching, aligning, and overlaying of three-dimensional protein structures based on a library of local protein motifs, the so-called SA. A distinctive feature and advantage of the proposed method is the use of graphical neural networking to transform the geometric and topological characteristics of the structure into SA sequences, which ensures the high accuracy of protein structure approximation, and the use of the well-known BLAST algorithm to search and align SA sequences. The synergy of these approaches ensures the high speed of searching for structures and high accuracy of structural comparisons. The SAFoldNet service can compete with the other available structural alignment search services in performance and, in some cases, can even outperform them.

The service has a modern and user-friendly web interface and is available to a wide range of researchers at https://psskb.org/similar-search link.

Overall, the use of SA for searching, aligning, and analyzing the evolution of protein structures is a promising avenue in structural biology and bioinformatics. Our future studies will be devoted to the development of a new version of SA, which will enable more accurate approximations of local structural motifs.

## Figures and Tables

**Table 1:** Comparison of the structure similarity search services of SAFoldNet and 3D-BLAST, PDBeFold, and RCSB PDB Search for the compliance of the detected structures with the query folds. CATH superfamilies for the search results were assigned using the FunFHMMer web server. The FunFHMMer web server provides domain-based protein functions (based on Gene Ontology) for query sequences based on the functional classification of the CATH-Gene3D resource.

PDB Code	Description	CATH Family	Service	Total	Accuracy of CATH Superfamily Assignment	Percent Coverage
2ko3	Ubiquitin	Beta-grasp (ubiquitin-like) 3.10.20	RCSB PDB Search	619	619	100%
			3D-BLAST	1034	629	60.8%
			PDBeFold	1999	1768	88.4%
			**SAFoldNet**	**2084**	**1970**	**94.5%**
2ocs	Pdz3 domain	Pdz3 domain 2.30.42	RCSB PDB Search	23	23	100%
			3D-BLAST	500	477	95.4%
			PDBeFold	688	655	95.3%
			**SAFoldNet**	**1000**	**935**	**93.5%**
1tit	Immunoglobulin	Immunoglobulin-like 2.60.40	RCSB PDB Search	13	13	100%
			3D-BLAST	123	86	68.2%
			PDBeFold	198	148	74.7%
			**SAFoldNet**	**237**	**199**	**83.9%**

**Table 2:** Training, test, and validation sets.

Total	Training Set (60%)	Test Set (30%)	Validation Set (10%)
88,440	53,064	26,532	8844

## Data Availability

This article contains supporting information. The code designed for SAFoldNet has been deposited in the open-access GitHub resource and is available at https://github.com/protdb/ABBNet (accessed on 3 June 2023).

## Supplementary Materials

The following supporting information can be downloaded at https://www.mdpi.com/article/10.3390/ijms241914439/s1.
