# Supplementary material for: SAFoldNet: A Novel Tool for Discovering and Aligning Three-Dimensional Protein Structures Based on a Neural Network
Source: Int J Mol Sci. 2023 Sep 22;24(19):14439. doi: 10.3390/ijms241914439 (PMC10572457; doi:10.3390/ijms241914439)
Supplement: Supplementary file 1 [file ijms-24-14439-s001.zip › ijms-2590064-supplementary.pdf]

Supplementary materials

Table S1. Model Training

Hyperparameter	Value (range)	Comments
Training epochs	46 (early stopping)	17.5h (192h)
Batch size	8	–
Learning rate	$1\times10^{-3} \rightarrow 1\times10^{-5}$	Reduce learning rate when validation loss has stopped improving
Optimizer	Adam	–
Loss function	Multiclass Cross Entropy	–
GPU	Nvidia A100	–

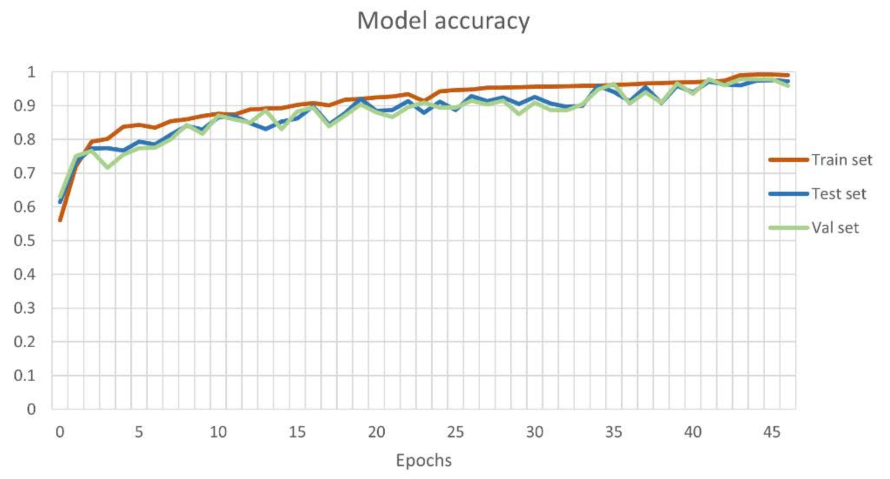

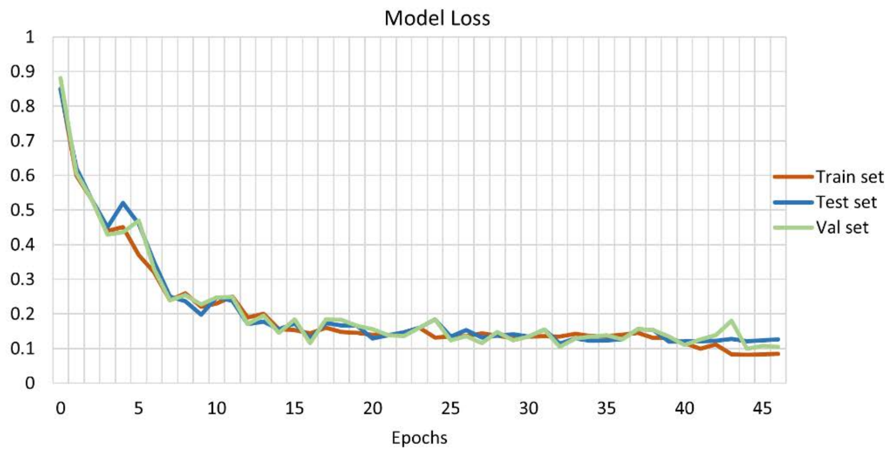

Figure S1. Model evaluation metrics.
